# Comparison of intrafractional motion with two frameless immobilization systems in surface‐guided intracranial stereotactic radiosurgery

**DOI:** 10.1002/acm2.13613

**Published:** 2022-04-19

**Authors:** Chunhui Han, Arya Amini, Jeffrey Y.C. Wong, Jieming Liang, Kun Qing, W. Tyler Watkins, Sean Zhang, Terence M. Williams, An Liu

**Affiliations:** ^1^ Department of Radiation Oncology City of Hope National Medical Center Duarte California USA

**Keywords:** intrafractional motion, stereotactic radiosurgery, surface‐guided radiosurgery

## Abstract

**Purpose/objectives:**

The aim of this study is to compare intrafractional motion using two commercial non‐invasive immobilization systems for linac‐based intracranial stereotactic radiosurgery (SRS) under guidance with a surface‐guided radiotherapy (SGRT) system.

**Materials/methods:**

Twenty‐one patients who received intracranial SRS were retrospectively selected. Ten patients were immobilized with a vacuum fixation biteplate system, while 11 patients were immobilized with an open‐face mask system. A setup margin of 1 mm was used in treatment planning. Real‐time surface motion data in 37 treatment fractions using the vacuum fixation system and 44 fractions using the open‐face mask were recorded by an SGRT system. Variances of intrafractional motion along three translational directions and three rotational directions were compared between the two immobilization techniques with Levene's tests. Intrafractional motion variation over time during treatments was also evaluated.

**Results:**

Using the vacuum fixation system, the average and standard deviations of the shifts were 0.01 ± 0.18 mm, ‐0.06 ± 0.30 mm, and  0.02 ± 0.26 mm in the anterior–posterior (AP), superior–inferior (SI), and left–right (LR) directions, and ‐0.02 ± 0.19°, ‐0.01 ± 0.13°, and 0.01 ± 0.13° for rotations in yaw, roll, and pitch, respectively; using the open‐face mask system, the average and standard deviations of the shifts were ‐0.06 ± 0.20 mm, ‐0.02 ± 0.35 mm, and 0.01 ± 0.40 mm in the AP, SI, and LR directions, and were 0.05 ± 0.23°, 0.02 ± 0.21°, and 0.00 ± 0.16° for rotations in yaw, roll, and pitch, respectively. There was a significant increase in intrafractional motion variance over time during treatments.

**Conclusion:**

Patients with the vacuum fixation system had significantly smaller intrafractional motion variation compared to those with the open‐face mask system. Using intrafractional motion techniques such as surface imaging system is recommended to minimize dose deviation due to intrafractional motion. The increase in intrafractional motion over time indicates clinical benefits with shorter treatment time.

## 1 INTRODUCTION

Stereotactic radiosurgery (SRS) plays an important role in the management of intracranial lesions including primary brain tumors and brain metastases from primary malignancies.^1^, ^2^ Accurate positioning and immobilization during treatments is essential to the success of SRS treatments. Traditionally, frame‐based fixation systems were first used in SRS treatments.^3^, ^4^ In a frame‐based fixation system, a head frame is rigidly fixated to the cranium of the patient and the target is localized with reference to the coordinates relative to the head frame. Later, frameless fixation systems were developed to allow fractionated treatments on medical linear accelerators (linacs) and to improve patient comfort.^5^ Examples of frameless fixation systems include biteplate devices^6^ and thermoplastic masks.^7^ In recent years, open‐face thermoplastic masks became available and provide greater comfort for patients undergoing intracranial SRS treatments. When a frameless fixation system is used, initial patient setup and target alignment are typically done with image guidance.^8^ However, when only pretreatment image guidance is performed, it is not known how consistent the patient immobilization with frameless fixation systems is in terms of intrafractional motion during treatment sessions.

In recent years, surface‐guided radiation therapy (SGRT) systems became available and provide real‐time monitoring of intrafractional motion during RT treatments.^9^, ^10^ At our institution, a commercial SGRT system was installed. In addition, a vacuum fixation biteplate system and a thermoplastic open‐face mask system were adopted as immobilization devices in intracranial SRS treatments. The purpose of this study was to evaluate and compare intrafractional motion of the cranial region in patients undergoing intracranial SRS treatments with the vacuum fixation biteplate system and the open‐face mask system, and to evaluate the benefits of the SGRT system for intracranial SRS treatments.

## 2 METHODS AND MATERIALS

A commercial SGRT system (the AlignRT system, Vision RT Ltd., London, UK) was installed in a medical linac treatment room at our institution. For this study, treatment record for all the SRS patients that were treated with surface guidance in recent 2 years at our institution are reviewed. Daily quality assurance and monthly calibration procedures were routinely performed on the SGRT system in this period according to manufacturer's recommendations. To eliminate uncertainty of motion detection by the SGRT system with couch rotations, patients that received treatments with non‐coplanar volumetric‐modulated arc therapy (VMAT) fields on the linac were excluded. A total of 21 patients were selected for this study. Ten of them were immobilized with a vacuum fixation system while the other 11 patients were immobilized with an open‐face thermoplastic mask system. Table 1 lists baseline characteristics for patients in this study.

**TABLE 1:** Baseline characteristics for patients in this study

Patient group	Vacuum fixation	Mask
Number of patients	10	11
Sex (male/female)	3/7	4/7
Age (years) (range)	55 ± 13 (30–69)	63 ± 9 (44–77)
Number of targets (range)	1.9 ± 1.5 (1–5)	2.4 ± 1.4 (1–5)
Treatment sessions	37	44

**FIGURE 1:**
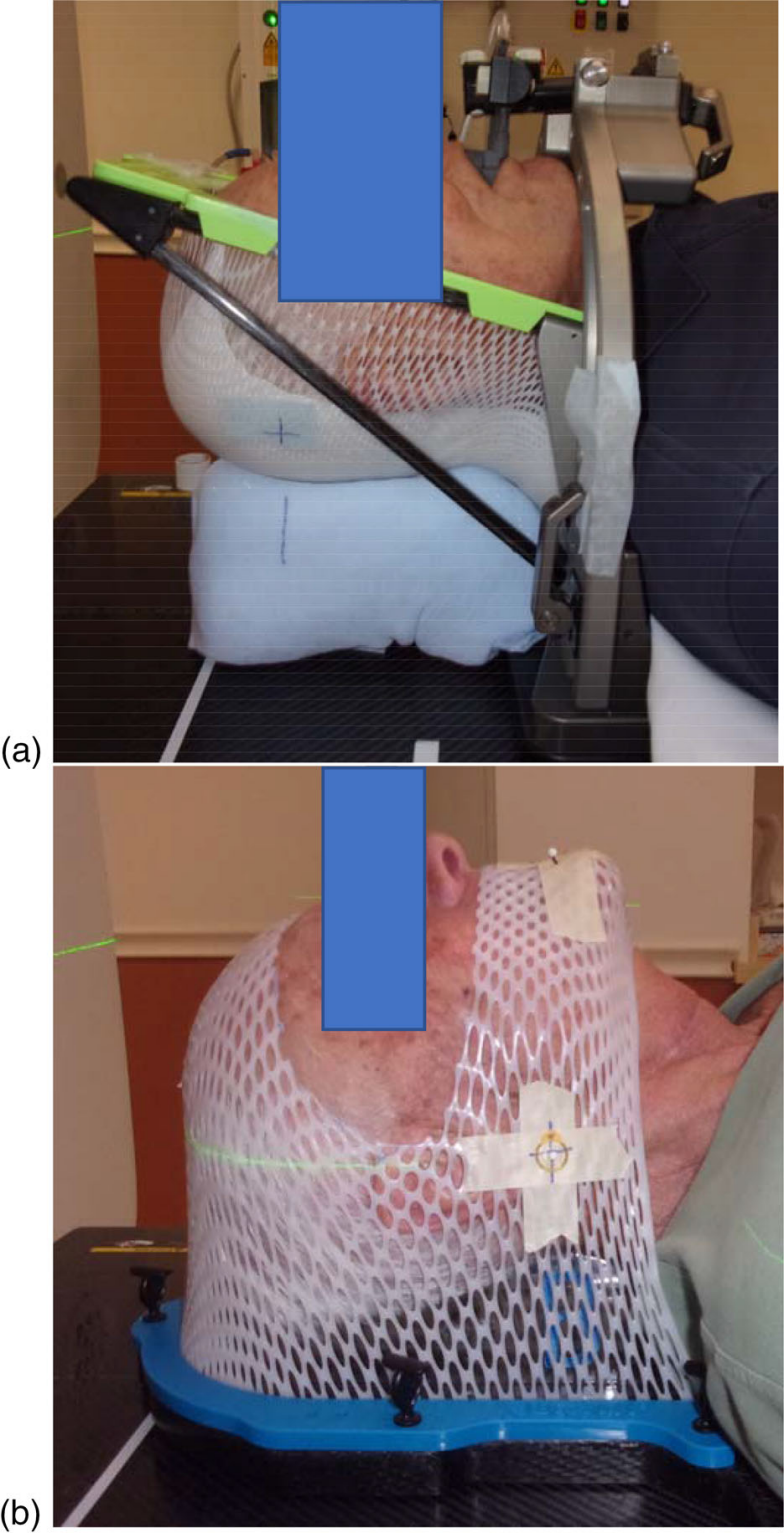
(a) Example setup with the vacuum fixation system and (b) example setup with the open‐face mask system

A commercial vacuum fixation biteplate system (the PinPoint system, Aktina Medical, Congers, NY, USA) was used for 10 patients in this study. The system uses a biteplate mouthpiece with vacuum suction for immobilization of the cranial structure. During computed tomography (CT) simulation, a biteplate mouthpiece was molded to conform to the upper dental contour and the hard palate of the patient. A vacuum suction tube was attached to the mouthpiece to ensure that the mouthpiece remains in tight contact with the upper denture and the hard palate both during CT simulation and in daily treatment delivery. Figure 1a shows one example of the setup with the biteplate mouthpiece.

The other 11 patients in this study were immobilized with an open‐face thermoplastic mask system (Klarity Medical Products USA, Heath, OH, USA). The open mask uses a thermoplastic material that is molded to conform to the patient head during CT simulation. As shown in Figure 1b, a mask opening extends longitudinally from the patient hairline to the middle of the philtrum and laterally to the level of auricularis anterior muscles. Three radiopaque triangulation markers were placed on the mask to define the origin of the coordinates used in the CT images.

All the CT simulations were performed with a slice thickness of 1.25 mm, a dimension of 50 cm for axial field‐of‐view, and axial image resolution of 512 × 512 pixels. The CT simulation images were transferred to a treatment planning system (TPS) (Eclipse version 15.5, Varian Medical Systems, Inc., Palo Alto, CA, USA) where target volumes were delineated by a radiation oncologist and critical organs were delineated by a dosimetrist or the radiation oncologist. A total of 60 intracranial gross target volumes (GTVs) were delineated. The planning target volumes (PTVs) were generated by adding a 1 mm isotropic margin to the GTVs. The PTV volumes ranged from 0.1 to 26.9 cm^3^ and the prescription dose to the PTVs ranged from 17 Gy in one fraction to 27 Gy in three fractions. A total of 40 treatment plans were created with each plan treating one to six PTVs. Each treatment plan only contained coplanar VMAT fields with the couch angle at 0°. A linear Boltzman transport algorithm (Acuros XB version 15.5, Eclipse TPS, Varian Medical Systems, Inc.) was used for dose calculation with a dose grid resolution of 1 mm. All the plans used 6 MV flattening filter free photon fields on a medical linac (TrueBeam‐STx, Varian Medical Systems, Inc.). A 120‐leaf multileaf collimator (MLC) (HD MLC, Varian Medical Systems, Inc.) was used for beam modulation, which had leaf widths of 2.5 mm for the central 32 leaf‐pairs and 5.0 mm for the peripheral 28 leaf‐pairs.

The AlignRT SGRT system uses three ceiling‐mounted camera pods for real‐time monitoring of surface motion. Each pod includes a projector and two image sensing cameras. During an SGRT treatment session, the projector creates a pseudo‐random speckle pattern onto the patient surface which is then picked up by the cameras to reconstruct a three‐dimensional surface. The user needs to define a region of interest (ROI), which is a subset of the skin surface area, to be used for surface motion tracking during treatments. During an SGRT session, the AlignRT system calculates the rigid motion of the current ROI position to a reference ROI position at a constant interval of about 0.36 s, thus providing real‐time surface positioning data.

At the beginning of each treatment session, therapists first set up the patient on the treatment couch by aligning room lasers to the radiopaque markers on the open mask or to the cross‐hairs on the alignment frame of the vacuum fixation system. Then the couch was shifted to move the linac isocenter to the treatment isocenter based on shifts from the user origin in the treatment plan. After the initial setup, a cone beam computed tomography (CBCT) scan was performed over the head region. The CBCT images were first registered to the planning CT images with an automatic registration algorithm using bony landmarks with six degrees of freedom including three translational directions and three rotational directions. The attending radiation oncologist subsequently reviewed image registration results and would make adjustment, if necessary, before approving the results. The approved image registration results were used to correct treatment couch position. As a result of image‐guided setup corrections, the true couch angles during beam delivery were not always the same as the planned couch angle of 0°. The average couch angle during beam delivery was ‐0.5 ± 1.1° (range: ‐3.3° to 1.5°, where a positive number indicates couch rotation in the clockwise direction). After the couch position was corrected and before beam delivery started, a reference ROI was captured by the SGRT system on a pre‐selected surface area, which extends from the hairline to the middle of the philtrum longitudinally and to the level of auricularis anterior muscles laterally. Beam delivery started promptly after the reference surface capture. During beam delivery, the SGRT system actively monitored the ROI position and calculated real‐time deviation of the ROI position from its reference position in the three translational directions and three rotational directions. In this study, the three translational directions are represented as *X*, *Y*, and *Z*, defined as shifts in the left–right (LR), anterior–posterior (AP), and superior–inferior (SI) directions, respectively; the three rotations are pitch, yaw, and roll, defined as rotations around the *X*, *Y*, and *Z* axis, respectively. Treatment would interrupt if the patient shifted more than 1 mm in any direction or rotated more than 1° in pitch, yaw, or roll. In most cases, shifts and rotations exceeded the tolerance limits only for a short period of time. When shifts and rotations fell back to the tolerance limits, beam delivery would resume automatically. In rare occasions, the patient might have a large motion which could not resolve within a reasonable time frame. Then therapists would repeat patient setup with CBCT‐based image guidance, acquire a new baseline surface image for the SGRT system, and resume treatment.

We retrieved all the real‐time intrafractional motion data at fixed intervals of about 0.36 s from all 37 treatment fractions for patients with the vacuum fixation system and from all 44 fractions for patients with the open‐face mask system. A total of 18 328 and 18 206 intrafractional motion data sets were analyzed for the vacuum fixation system and the open‐face mask system, respectively. The Jarque–Bera normality test was performed on intrafractional motion data along each direction to evaluate if intrafractional motion was normally distributed. To illustrate deviation from a normal distribution, a normal distribution curve was fitted to intrafractional motion data in each of the translational and rotational directions. In addition, the skewness and kurtosis of each distribution was calculated to show deviation from a normal distribution. The absolute translational shifts (*L*) were also calculated, which was defined as: $L\;=\sqrt{X^{2}+Y^{2}+Z^{2}}\;$, with *X*, *Y*, and *Z* being shifts in the LR, AP, and SI directions, respectively. To test whether using the open‐face mask system led to greater variation in intrafractional motion than the vacuum fixation system, Levene's test was used to test whether the variances in the two groups of data are equal. Statistical analysis in this study was performed with a data analysis software system (Excel version 2102, Microsoft Corp., Redmond, WA, USA).

## 3 RESULTS

**FIGURE 2:**
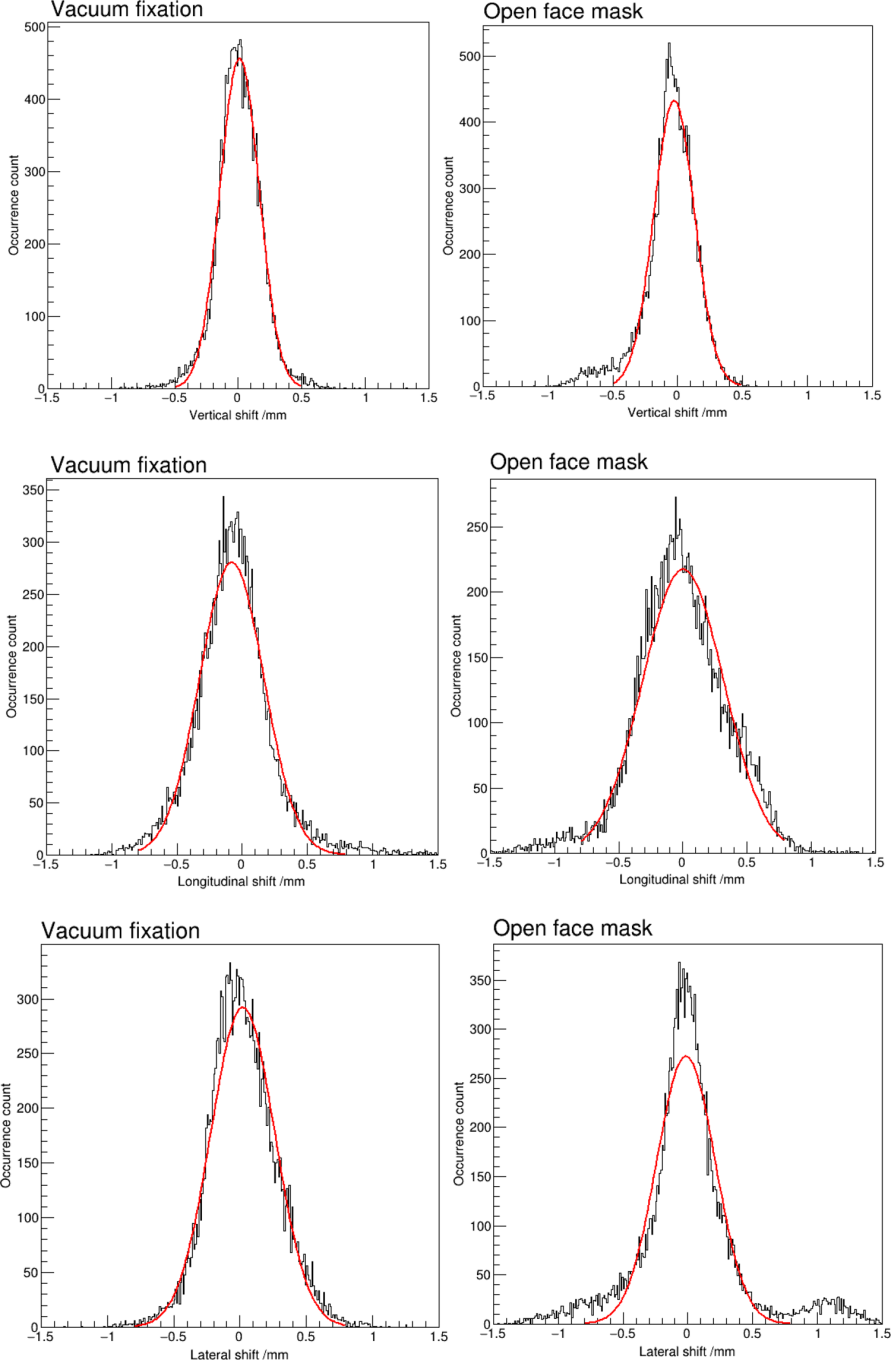
Distribution of intrafractional motion along each translational direction with each of the two immobilization techniques. The red curve on each plot is the fitted normal distribution curve

Intrafractional motion data were retrieved in 37 treatment sessions using the vacuum fixation system and 44 treatment fractions using the open‐face mask system. The average beam on time was 176.4 ± 88.5 s and 166.5 ± 76.4 s for treatment sessions using the vacuum fixation system and the mask system, respectively. Figure 2 shows distributions of intrafractional translational shifts in each of the three orthogonal directions for patients with the vacuum fixation system and the open‐face mask system, respectively. Table 2 lists statistics of intrafractional motion in each translational direction. With the vacuum fixation system, the average shifts and standard deviations were 0.01 ± 0.18 mm, ‐0.06 ± 0.30 mm, and 0.02 ± 0.26 mm in the AP, SI, and LR directions, respectively; with the open‐face mask system, the standard deviations were ‐0.06 ± 0.20 mm, ‐0.02 ± 0.35 mm, and 0.01 ± 0.40 mm in the AP, SI, and LR directions, respectively. Table 2 also lists two‐tailed *p*‐values from Levene's tests for variances of intrafractional shifts in each direction between the two groups of patients and the results showed statistically significant difference between the two groups of data. Magnitude of shifts along each orthogonal direction was evaluated for all the instantaneous data points in this study. With the vacuum fixation system, intrafractional motion was less than 1 mm in any direction 99.1% of the time, while with the open‐face mask system, intrafractional motion was less than 1 mm in any direction 94.2% of the time. Beam delivery was paused when the translational shift was greater than 1 mm in any direction, which led to treatment pauses in 14 treatment sessions with the vacuum fixation system and in 12 treatment sessions with the open‐face mask system. Based on Jarque–Bera normal test results, there was statistically significant deviation from a normal distribution with intrafractional motion data along each translational direction using either immobilization technique. To illustrate the difference from a normal distribution, a normal distribution curve was fitted to each distribution and was plotted as the red curve in Figure 2. Table 3 lists values of the fitted normal distribution curve parameters. With the vacuum fixation system, the average and standard deviations of the fitted normal distribution curves were 0.01 ± 0.16 mm, 0.02 ± 0.24 mm, and ‐0.08 ± 0.25 mm in the lateral, AP, and SI directions, respectively. With the open‐face mask system, the standard deviations of the fitted normal distribution curves were ‐0.03 ± 0.16 mm, ‐0.01 ± 0.23 mm, and 0.00 ± 0.32 mm, respectively. In addition to the normal distribution function fitting, the skewness and kurtosis of each distribution plot was also calculated, and the results are listed in Table 3.

**TABLE 2:** Statistics of intrafractional motion in translational shifts and rotations

	Average ± standard deviation		Range of motion		
Direction	Vacuum fixation	Open‐face mask	Vacuum fixation	Open‐face mask	*p*‐Value
AP (mm)	0.01 ± 0.18	‐0.06 ± 0.20	(‐1.22, 2.12)	(‐1.10, 0.61)	<0.01
SI (mm)	‐0.06 ± 0.30	‐0.02 ± 0.35	(‐1.16, 4.14)	(‐1.50, 1.84)	<0.01
LR (mm)	0.02 ± 0.26	0.01 ± 0.40	(‐1.77, 1.25)	(‐1.78, 1.83)	<0.01
Yaw (°)	‐0.02 ± 0.19	0.05 ± 0.23	(‐1.00, 0.80)	(‐0.75, 1.21)	<0.01
Roll (°)	‐0.01 ± 0.13	0.02 ± 0.21	(‐0.68, 0.90)	(‐0.66, 1.03)	<0.01
Pitch (°)	0.01 ± 0.13	0.00 ± 0.16	(‐0.60, 2.20)	(‐0.53, 0.63)	<0.01

*Note*: The two‐tailed *p*‐values are from Levene's tests for variances between the two groups of patients.

Abbreviations: AP, anterior–posterior; LR, left–right; SI, superior–inferior.

**TABLE 3:** Statistics for the fitted normal distribution functions for shifts and rotations in each direction

Direction	Immobilization	*μ* (mm)	*σ* (mm)	Skewness (mm)	Kurtosis (mm)
LR	Vacuum fixation	0.01	0.16	0.01	2.93
	Mask	‐0.03	0.16	‐0.99	2.09
AP	Vacuum fixation	0.02	0.24	0.12	0.95
	Mask	‐0.01	0.23	0.64	2.67
SI	Vacuum fixation	‐0.08	0.25	0.87	5.17
	Mask	0.00	0.32	‐0.22	0.87
		*μ* (°)	*σ* (°)	Skewness (°)	Kurtosis (°)
Pitch	Vacuum fixation	0.01	0.11	0.72	7.67
	Mask	0.00	0.16	0.11	0.05
Yaw	Vacuum fixation	‐0.01	0.18	‐0.23	1.52
	Mask	0.03	0.19	0.69	2.40
Roll	Vacuum fixation	0.00	0.10	‐0.91	2.37
	Mask	0.02	0.19	0.20	0.91

*Note*: *μ* and *σ* are the mean and standard deviation of the normal distribution function that was used to fit the data.

Abbreviations: AP, anterior–posterior; LR, left–right; SI, superior–inferior.

**FIGURE 3:**
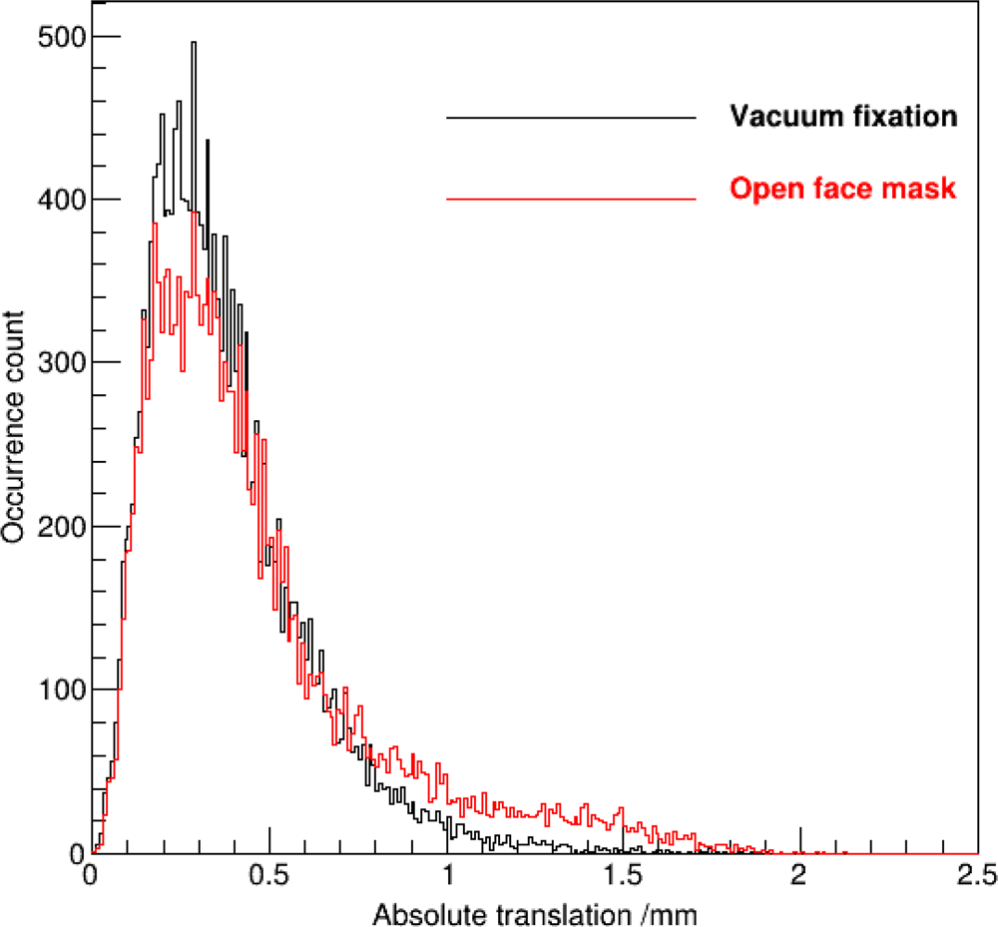
Distribution of the absolute translational intrafractional shifts with each of the two immobilization techniques

Figure 3 shows the distribution of absolute translational shifts for patients with each immobilization technique. The absolute translational shift was greater than 1 mm in 1.9% of the time with the vacuum fixation system and in 8.6% of the time with the open‐face mask system.

**FIGURE 4:**
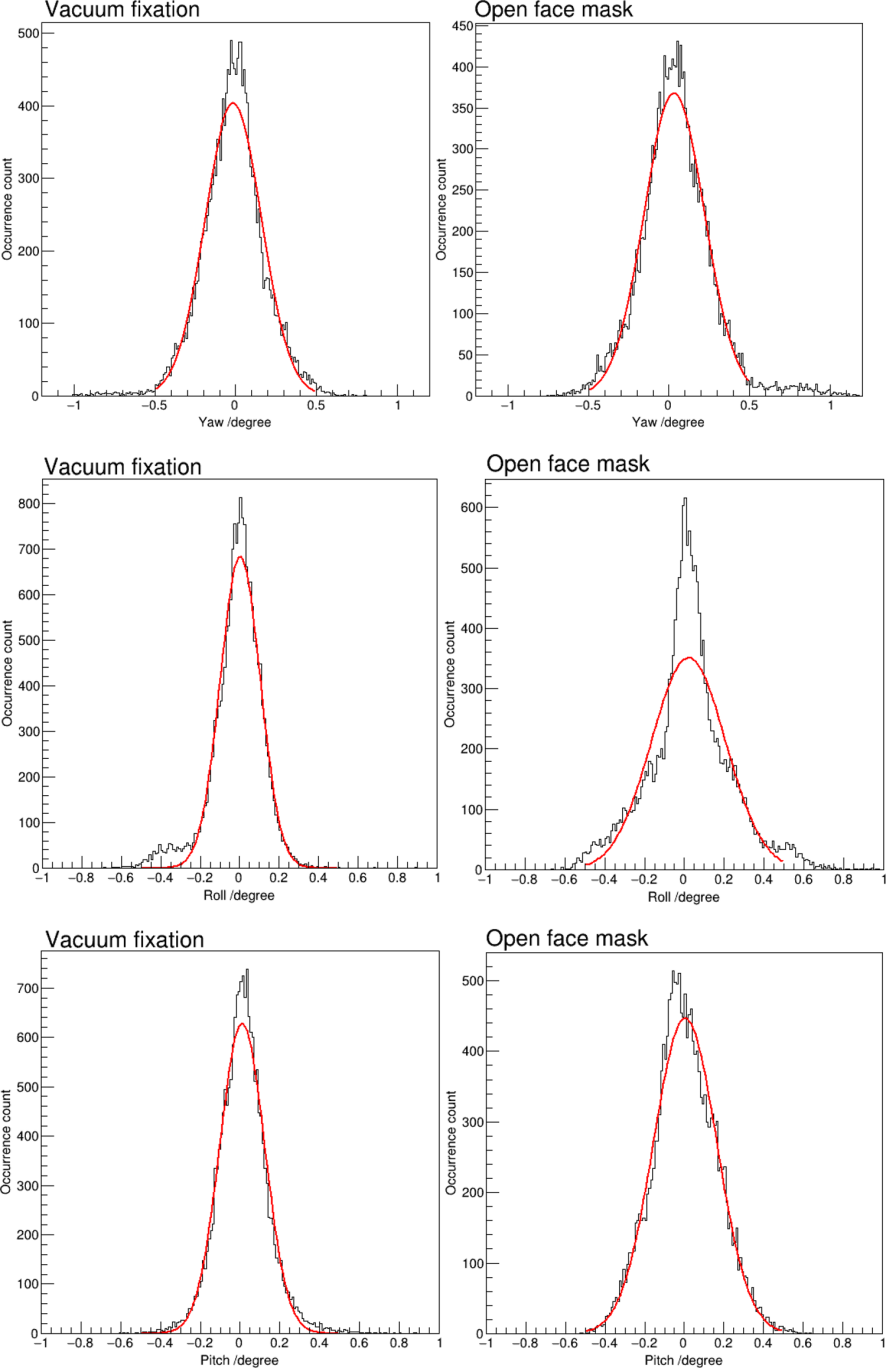
Distribution of intrafractional motion along each rotational direction with each of the two immobilization techniques. The red curve on each plot is the fitted normal distribution curve

Figure 4 shows distributions of intrafractional rotations in each of the three orthogonal directions for patients with the vacuum fixation system and the open‐face mask system, respectively. Table 2 lists statistics of intrafractional rotations in each rotational direction. With the vacuum fixation system, the average and standard deviations were ‐0.02 ± 0.19°, ‐0.01 ± 0.13°, and 0.01 ± 0.13° in yaw, roll, and pitch, respectively; with the open‐face mask system, the average and standard deviations were 0.05 ± 0.23°, 0.02 ± 0.21°, and 0.00 ± 0.16° in yaw, roll, and pitch, respectively. Table 2 also lists two‐tailed *p*‐values from Levene's tests for variances with rotations in each direction between the two groups of patients and the results showed statistically significant difference between the two groups of data. With the vacuum fixation system, intrafractional rotation was less than 0.5° in any direction in 98.1% of the time. While with the open‐face mask system, intrafractional rotation was less than 0.5° in any direction in 93.4% of the time. Based on Jarque–Bera normal test results, there was statistically significant deviation from a normal distribution with intrafractional motion data along each rotational direction using either immobilization technique. To illustrate the difference from a normal distribution, a normal distribution curve was fitted to each distribution and was plotted as the red curve in Figure 4. Table 3 lists values of the fitted normal distribution curve parameters. In addition to the normal distribution function fitting, the skewness and kurtosis of each distribution plot was also calculated, and the results are listed in Table 3.

**FIGURE 5:**
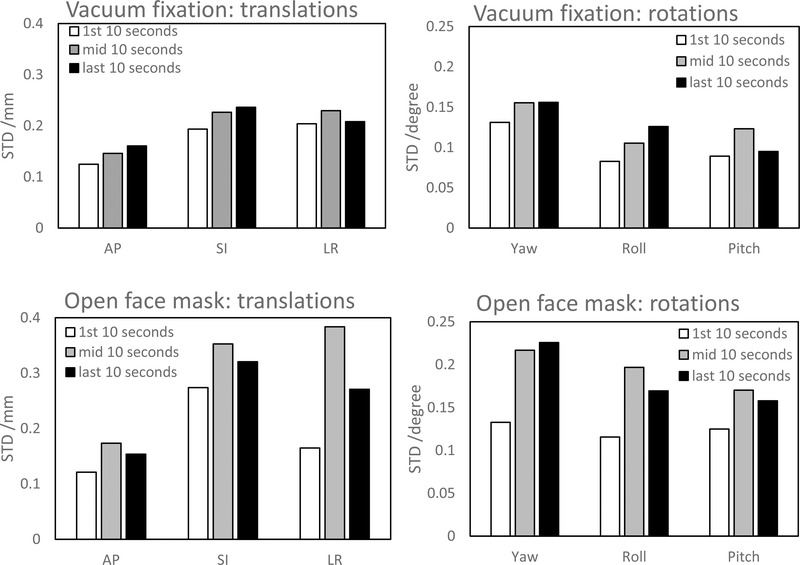
Variation in standard deviations of intrafractional motion along each translational and rotational direction at three segments in time during treatments. STD: standard deviation

Figure 5 shows standard deviations of intrafractional motion in each of the translational and rotational directions during each of the following three segments in time during treatment sessions: the initial 10 s after beam on, the 10 s around the middle of each treatment session, and in the last 10 s of each treatment session. The average time between the first 10‐s time segment and the 10‐s time segment in the middle of the treatment was 83.20 ± 44.26 s for patients with the vacuum fixation system and was 78.26 ± 38.21 s for patients with the open‐face mask system. The same numbers applied to the average time between the 10‐s time segment in the middle of the treatment and the last 10‐s time segment. Compared to the first 10 s, the standard deviations for all the translational and rotational directions were higher in the middle and end time segments of the treatment sessions. The highest relative increase was 28.8%, 22.0%, 12.6%, 19.0%, 52.3%, and 38.1% in the AP, SI, LR, yaw, roll, and pitch directions when the vacuum fixation system was used; the highest relative increase was 43.1%, 28.8%, 132.9%, 70.0%, 70.3%, and 36.3% in the AP, SI, LR, yaw, roll, and pitch directions when the open‐face mask system was used. Levene's tests were performed on the differences in the variances of intrafractional motion between different time segment along each direction. The increase in variance from the initial 10 s was statistically significant (two‐tailed *p*‐value < 0.05) in each direction with at least one of the subsequent two segments in time.

To evaluate if correlation exists between motions in different translational or rotational directions using either immobilization technique, both Pearson's correlation coefficients and Spearman's rank correlation coefficients were assessed between motions along each pair of translational axes and between rotations along each pair of rotational axes for the data using each of the two immobilization techniques. Both the absolute values of Pearson's correlation coefficients and the absolute values of Spearman's rank correlation coefficients were all within 0.3, indicating that there was no strong correlation between any pair of translational motion variables or between any pair of rotational motion variables when either the vacuum fixation system or the open‐face mask system was used.

## 4 DISCUSSIONS

Linac‐based SRS is a widely used treatment modality in the management of intracranial malignancies. In this study, we evaluated the efficacies of two commercial frameless immobilization systems to limit intrafractional motion in intracranial SRS treatments using a surface‐guidance system. Compared to those treated with the vacuum fixation system, patients with the open‐face mask system had significantly greater variation in intrafractional motion with both translations and rotations. On the other hand, the use of the SGRT system allowed real‐time monitoring of intrafractional motion and treatment pause when intrafraction motion exceeded preset limits. The results from this study can be used to help clinicians choose the proper immobilization technique for intracranial SRS, determine the setup margins to be used in treatment planning, and evaluate whether intrafractional motion should be actively monitored in clinical intracranial SRS treatments.

To minimize risks for marginal failures and recurrences in addition to treatment‐related toxicities including radionecrosis, it is important to reduce both interfractional and intrafractional motions during intracranial SRS treatments so that a small setup margin can be used in treatment planning and dose is delivered accurately to the target volume. Intrafractional motion depends on the immobilization technique used and can be monitored by different technologies including stereoscopic X‐ray imaging, megavoltage imaging, infrared sensors, pressure sensors, and surface imaging.^11^, ^12^, ^13^, ^14^, ^15^ Compared to imaging methods utilizing ionizing beams, surface imaging does not give additional dose to the patient and could achieve sufficient accuracy in detection of patient positions.^9^ Wiant et al.^16^ compared surface imaging with orthogonal radiographic imaging using an anthropomorphic head phantom. They found that the surface imaging system achieved similar accuracy with a room mounted X‐ray imaging at 72 phantom positions. Li et al.^17^ also evaluated the accuracy of an optical surface imaging system and found that the system could achieve an accuracy of 0.1 mm with uncertainty of ±0.1 mm with a head phantom. Our clinical experience showed that the use of a surface imaging system did not introduce significantly overhead with clinical workflow as the SGRT system monitors intrafractional motion independent of beam delivery and would pause the beam only when intrafractional motion exceeds preset limits.

This study also showed difference in intrafractional motion variation along different directions. As shown in both Figure 2 and Table 2, the standard deviation of translational motion was the smallest in the AP direction with both the vacuum fixation system and the open‐face mask system. However, shifts greater than 1 mm occurred in the SI direction with the vacuum fixation system in a small but noticeable number of the time, and shifts greater than 1 mm occurred in both the SI and RL directions with the open‐face mask system in a small but noticeable number of the time. With a setup margin of 1 mm used in treatment planning, such deviations would lead to insufficient dose delivered to the target volumes without active tracking of intrafractional motion and beam hold based on motion tracking. In the clinical intracranial SRS treatments at our institution, the SGRT system was configured to pause beam delivery automatically when the translational shift was greater than 1 mm in any direction. Treatment delivery would resume only when the translation shifts were within 1 mm from the reference position before the start of each treatment session. The overall beam hold time was 1.9% and 8.6% of the total treatment time with the vacuum fixation system and with the open‐face mask system, respectively, indicating that the overall treatment time was increased by 1.9% and 9.4% with the vacuum fixation system and the open‐face mask system, respectively, due to detected intrafractional motion.

Intrafractional rotations contribute to delivery accuracy when targets are located away from the treatment isocenter. A rotation of 1° around the isocenter will lead to a 1‐mm shift for an object at 6 cm from the isocenter, and the shift will increase to almost 2 mm at 10 cm from the isocenter. When multiple intracranial targets are treated with one isocenter, adequate immobilization technique should be used to minimize intrafractional rotations. Both the vacuum fixation system and the open‐face mask system limited intrafractional rotations within 0.5° at any direction in 98.1% and 93.4% of the time, respectively. Of note, rotations greater than 0.5° occurred in the yaw and roll directions with the open‐face mask system in a small but noticeable number of the time as shown in Figure 4. When a treatment plan includes targets at a large distance from the isocenter, proper immobilization techniques should be applied and intrafractional rotations should be monitored.

Previous studies investigated correlation between intrafractional motion variation and the beam on time during the treatment sessions. Wang et al.^18^ evaluated intrafractional real‐time X‐ray images taken during intracranial SRS on a robotic radiosurgery system and found consistent increase in positioning deviation over time. Using an infrared marker system, MacDonald et al.^19^ found that that rate of intrafractional motion exceeding preset limits steadily increased with treatment time, indicating a benefit from minimizing treatment time. The results from this study showed that the intrafractional motion variation increased significantly during treatments compared to the beginning of treatments, indicating potential clinical benefits from shorter treatment time. When patients receive intracranial SRS with extended treatment time, proper motion monitoring technique should be used.

There are limitations in this study. First, all the data were from a single institution. The results may be affected by the clinical experience of the treatment team in using each type of the immobilization systems. Second, there could be systematic and random errors with the SGRT system in motion detection. On the other hand, since patients with the vacuum fixation system were treated during the same period as those with open‐face masks in this study, systematic or random errors with the AlignRT system would have had equal impact on the intrafractional motion data for both groups of patients, limiting impact to the results and conclusions in this study.

## 5 CONCLUSIONS

Patients with the vacuum fixation system had significantly smaller translational and rotational intrafractional motion variation compared to those with the open‐face mask system. Occasional positioning deviations greater than 1 mm occurred with both immobilization systems. The use of intrafractional motion techniques such as surface imaging system is recommended to minimize dose deviation due to intrafractional motion. Shorter treatment time provides clinical benefits due to the increase in intrafractional motion over treatment time.

## AUTHOR CONTRIBUTIONS

Chunhui Han performed data acquisition, data analysis, and drafting of the manuscript. Arya Amini, Jeffrey Y.C. Wong, Jieming Liang, Kun Qing, William Watkins, Sean Zhang, Terence M. Williams, and An Liu contributed to data analysis methodology, the interpretation of data, critical review of the manuscript, and final approval of the version to be published. All the authors agreed to be accountable for all aspects of the work in ensuring that questions related to the accuracy of integrity of any part of the work are appropriately investigated and resolved.

## CONFLICT OF INTEREST

None.
